# Prevalence of latent tuberculosis infection and predictive factors in an urban informal settlement in Johannesburg, South Africa: a cross-sectional study

**DOI:** 10.1186/s12879-016-1989-x

**Published:** 2016-11-08

**Authors:** Jabulani R. Ncayiyana, Jean Bassett, Nora West, Daniel Westreich, Eustasius Musenge, Michael Emch, Audrey Pettifor, Colleen F. Hanrahan, Sheree R. Schwartz, Ian Sanne, Annelies van Rie

**Affiliations:** 1 Department of Epidemiology, Gillings School of Global Public Health, University of North Carolina at Chapel Hill, Chapel Hill, NC 27599 USA; 2 Division of Epidemiology and Biostatistics, School of Public Health, Faculty of Health Sciences, University of the Witwatersrand, 29 Princess of Wales Terrace, Johannesburg, 2193 South Africa; 3 Witkoppen Health and Welfare Centre, 105 William Nicol Drive, Fourways, Johannesburg, 2055 South Africa; 4 Department of Epidemiology, Bloomberg School of Public Health, Johns Hopkins University, 615 N. Wolfe Street, Baltimore, MD 21205 USA; 5 Clinical HIV Research Unit, Department of Medicine, University of the Witwatersrand, Perth Road, Auckland Park, Johannesburg, 2092 South Africa; 6 Department of Epidemiology and Social Medicine, Faculty of Medicine and Health Sciences, University of Antwerp, Campus Drie Eiken, University Square, Wilrijk, Antwerp, 2610 Belgium

**Keywords:** South Africa, LTBI, Prevalence, ARI, Risk factors, Urban population

## Abstract

**Background:**

South Africa has one of the highest burdens of latent tuberculosis infection (LTBI) in high-risk populations such as young children, adolescents, household contacts of TB cases, people living with HIV, gold miners and health care workers, but little is known about the burden of LTBI in its general population.

**Methods:**

Using a community-based survey with random sampling, we examined the burden of LTBI in an urban township of Johannesburg and investigated factors associated with LTBI. The outcome of LTBI was based on TST positivity, with a TST considered positive if the induration was ≥5 mm in people living with HIV or ≥10 mm in those with unknown or HIV negative status. We used bivariate and multivariable logistic regression to identify factors associated with LTBI

**Results:**

The overall prevalence of LTBI was 34.3 (95 % CI 30.0, 38.8 %), the annual risk of infection among children age 0–14 years was 3.1 % (95 % CI 2.1, 5.2). LTBI was not associated with HIV status. In multivariable logistic regression analysis, LTBI was associated with age (OR = 1.03 for every year increase in age, 95 % CI = 1.01–1.05), male gender (OR = 2.70, 95 % CI = 1.55–4.70), marital status (OR = 2.00, 95 % CI = 1.31–3.54), and higher socio-economic status (OR = 2.11, 95 % CI = 1.04–4.31).

**Conclusions:**

The prevalence of LTBI and the annual risk of infection with *M. tuberculosis* is high in urban populations, especially in men, but independent of HIV infection status. This study suggests that LTBI may be associated with higher SES, in contrast to the well-established association between TB disease and poverty.

## Background

Tuberculosis (TB) remains a significant public health problem worldwide with an estimated 9.6 million cases and 1.5 million deaths in 2014 [1]. In 2014, South Africa had the second highest burden of TB in the African region and was ranked sixth among the 22 countries classified by the World Health Organization (WHO) as high TB burden countries [1]. More South Africans died of TB, predominantly HIV-associated TB, than any other disease [2]. These statistics suggest that the current TB control strategy is unable to control the TB epidemic in South Africa, which is fueled by both progression from LTBI to active disease, in large part due to HIV co-infection, and ongoing transmission of *Mycobacterium tuberculosis (M. tuberculosis)* [3].

Globally, about 2.6 billion people are infected with *M. tuberculosis*, representing a large reservoir of people at risk of progression to active TB disease [1, 4–7]. About 5–10 % of people with LTBI progress to active TB disease in their lifetime, the majority within 2 years of infection [8]. Those at highest risk of progression to active TB disease are young children and immunocompromised individuals [7, 9, 10]. To date, studies of the burden of LTBI in South Africa have mainly focused on high-risk populations such as young children, adolescents, household contacts of TB cases, people living with HIV, gold miners and health care workers [11–16]. These studies observed LTBI prevalence ranging from 26 % up to 89 %. The only community-based study, performed in an urban township of Cape Town, observed a very high (88.0 %) LTBI prevalence rate, but the study was limited to healthy HIV-negative individuals [17].

The goal of this study was to describe the burden of LTBI in a representative sample of all residents of an urban South Africa township and determine factors associated with LTBI.

## Methods

### Study site and Study population

The study was conducted in Diepsloot, a densely populated, urban township located in northern Johannesburg, South Africa. The community covers an area of 12 km^2^ and has an estimated population of 136,289, corresponding to a very high population density of 11,357 people/km^2^ [18]. The area is typical of urban South African townships, consisting of informal settlements with a mix of high-density shacks and government-subsidized brick houses. According to the 2006 Johannesburg Poverty and Livelihoods Study, Diepsloot is one of the poorest urban informal settlements in Johannesburg [19].

The analysis represents a sub-study of a large community-based household health survey conducted between May 2013 to March 2014 using a random sampling framework. Geographic coordinates were generated from an aerial map of the 13 digital geo-referenced extensions of the township. The township extensions are designated areas (neighbourhoods) within the township. Geographic coordinates were randomly selected within each extension and the number of coordinates per extension was proportional to the population density of the extension. The randomly selected coordinates were then located by the study team using a hand-held geographic positioning system (GPS) device (eTrex 10, Garmin). The household nearest to but within 30 m of each randomly selected geographic coordinate was eligible for study participation. If multiple households were equidistant from the geo-coordinate, households within the same distance were numbered, and then the survey team randomly selected one household using a random number generator. Following this method, survey teams approached 2006 households. Households, where no-one could be found home despite up to five repeat visits, were considered missing and not replaced.

At the time of the home visit, the exact latitude and longitude coordinates of the house were geocoded. When the household member agreed for the household to participate in the survey, all household members were enumerated. one of the enumerated adult (≥15 years) household members was randomly selected for study participation using the Kish grid method [20]. To be an eligible household member, each adult had to sleep in the household at least 1 night per week*.* This procedure was implemented to avoid the selection bias that would have occurred had the adult household member at home at the time of the survey been systematically selected for study participation. If the adult household member selected for study participation was not home, then the survey team made up to 4 attempts before the household member was considered unreachable. Selected adults who could not be reached were not replaced. All childhood household members were invited to participate in a health assessment if the selected adult household member consented for their study participation. If a child <15 was not in the household at the time the selected adult participant was interviewed, no return home visits were made for the child.

Using a structured questionnaire in English, Sesotho or IsiZulu, data on socio-demographics and household characteristics, education and employment, history of TB or contact with TB, and alcohol and smoking habits were collected from all adult participants. A health assessment was performed in all adult and child participants. Weight and height were measured, and blood was collected for haemoglobin and HIV testing by a trained lay HIV counsellor. Participants were assessed for symptoms of active TB and a tuberculin skin test (TST) was placed by a trained nurse. A quantity of 0.1 ml (5TU) of purified protein derivative (PPD) (Aplisol and Tubersol) was injected in the fore arm; the size of induration was read 48 to 72 h later. Because of a high rate of adverse events in HIV negative individuals, including blistering and ulceration, the ethics committee overseeing the study recommended in October 2014 to restrict the placement of TST to HIV positive individuals.

### Study variables

The outcome of LTBI was based on TST positivity, with a TST considered positive if the induration was ≥5 mm in people living with HIV or ≥10 mm in those with unknown or HIV negative status [21].

Individual covariates included age (<15, 15–24, 25–34, 35–44 or ≥45 years); sex (male or female), HIV status (positive or negative), Body Mass Index (BMI; underweight/normal if BMI ≤18.5–24.9 kg/m^2^, overweight if BMI 25–29.9 kg/m^2^, or obese if BMI ≥30 kg/m^2^) presence of anaemia (with anaemia defined as haemoglobin value below 13.0 g/dl for men, <12.0 g/dl for women and children aged 12 to15 years, <11.0 g/dl or children under 5 years, or <11.5 g/dl for children aged 5 to12 years; all down-adjusted by 0.65 g/dl because of altitude), [22] education (primary or less vs. secondary or higher); marital status (living with partner or not living with a partner); employment status (unemployed or employed); household contact with TB (yes or no); smoking status (ever or never), and alcohol consumption (yes or no).

The household-level covariates included were household socioeconomic status (SES), household ventilation and household exposure to smoking. Household SES was calculated as a composite index developed by factor analysis based on household ownership of durable goods (car, motorcycle, bicycle, refrigerator, television, radio, and mobile phone), house ownership, source of drinking water, and type of toilet facilities [23, 24]. Household SES indices were categorized into tertiles of highest, median and lowest household SES. Household ventilation was defined based on the frequency household members sleep with the window open (always, only when warm enough, never, no windows in the house), household exposure to secondary smoking as (yes or no).

We created 20 neighbourhoods from the 13 extensions by further subdividing 5 largest extensions of Diepsloot township. Neighbourhood-level factors included neighbourhood SES which was obtained by summarizing household SES by 20 neighbourhood, population density defined as the number of people per square kilometre (low, medium or high) and household density defined as the number of households per square kilometre (low, medium or high). Population and household density data were retrieved from the 2011 South African census as disseminated by Statistics South Africa (STATSSA) using the SuperCROSS software [18].

### Statistical analysis

LTBI prevalence was calculated by dividing the number of participants with a positive TST by the total number of participants with a TST results and 95 % confidence intervals (95 % CI) were estimated. Annual risk of infection (ARI) with *M. tuberculosis* in children age 0 to 14 years was calculated using the formula *ARI* = 1 − (1 − *P*)^1/*a*^; where P is the observed prevalence of LTBI, and *a* the mean age of participating children [25, 26].

We opted for a multilevel (hierarchical) structure of our data with individuals and households (first level) nested into 20 township neighbourhoods (second level). We calculated the intraclass correlation coefficient (ICC) to assess the magnitude of variability due to the covariates at the neighbourhood level in order to determine whether multilevel logistic models were appropriate [27–29]. The ICC was calculated by fitting a “null model” using the Stata command “*gllamm”* within the generalized linear latent and mixed methods framework, for binary response outcome [30].

We used bivariate and multivariable logistic regression to identify factors associated with LTBI. Starting from a full model with all potential predictors, we employed a stepwise backward elimination approach removing the least significant factor one at a time until all remaining factors were significant. We repeated the model building procedures using stepwise forward selection to check whether this yielded the same final model. Associations between predictors and LTBI are summarized in odds ratio (OR) along with 95 % CIs. A *p*-value of <0.05 was considered statistically significant. Data analysis was conducted using Stata version 13.1 (Stata Corp, College Station, TX).

## Results

### Study participants

**Fig. 1:**
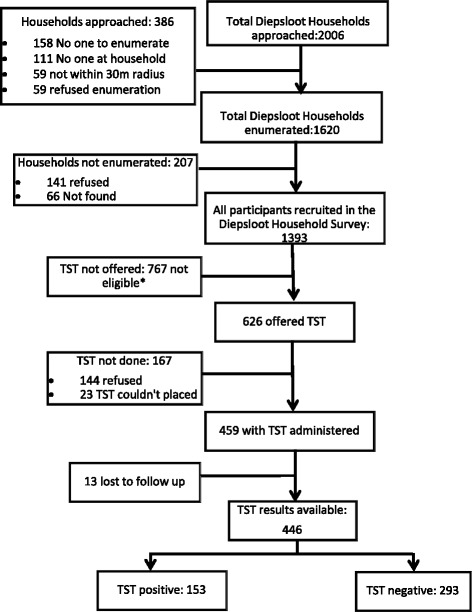
Flow chart of study participants. *These participants were not eligible for TST according to SA National TB guidelines

Of the 2006 randomly selected households, 1620 could be enumerated. Of the 1620 randomly selected adults, 1581 (97.6 %) could be contacted and 1230 agreed to participate (Fig. 1). In addition, 169 children living in the same household as the participating adult were enrolled. TST was offered to 626 participants (all participants until October 2014, only HIV positive individuals thereafter). Of these, 144 refused and TST was not placed in 23. Of the 459 participants in whom a TST was placed, TST was read in 446 (97 %), the remaining 13 could not be traced within 48–72 h of TST placement.

Among the 446 participants with TST result, mean age was 35 years, 11 % were 0 to 15 years of age, 17 % were 15 to 24 years, 33 % were 25 to 34 years, 18 % were 35 to 34 years and 21 % were 45 years or older (Table 1). Sixty percent were female, 44 % were married or living with a partner, two thirds (67 %) were unemployed and the majority (72 %) had at least some secondary education. Self-report of smoking (26 %) and alcohol use (37 %) was relatively low. Overall, 18 % of the 446 participants with TST result were HIV positive, 35 % were anaemic, 23 % were underweight and 27 % obese. Only 6 % of participants reported a history of contact with a TB case. Almost all (93.4 %) participants either lived in a house without windows or never slept with windows open and 20 % were exposed to household secondary smoking.

**Table 1:** Characteristics of 446 participants with positive and negative TST results

Characteristics		TST Positive *N* (row %)	TST Negative *N* (row %)	Total *N* (column %)
Age in Years median (IQR)		35 (27–45)	29 (22–38)	32 (23–41)
Age group	0–14	9 (18.7)	39 (81.3)	48 (10.8)
	15–24	20 (26.3)	56 (73.7)	76 (17.0)
	25–34	45 (30.6)	102 (69.4)_	147 (33.0)
	35–44	37 (45.1)	45 (54.9)	82 (18.4)
	≥45	42 (45.2)	51 (54.8)	93 (20.8)
Sex	Female	86 (32.2)	181 (67.8)	267 (60.4)
	Male	65 (37.1)	110 (62.9)	175 (39.6)
HIV status	Positive	23 (32.9)	47 (67.1)	70 (18.1)
	Negative	115 (36.3)	202 (63.7)	317 (81.9)
BMI (kg/m^2^) median (IQR)		25 (21–29)	23 (20–29)	24 (20–29)
BMI categories	Normal/underweight	70 (34.8)	131 (65.2)	201 (49.9)
	Overweight	39 (41.5)	55 (58.5)	94 (23.3)
	Obese	37 (34.3)	71 (65.7)	108 (26.8)
Anaemia	Anaemic	51 (32.3)	107 (67.7)	158 (35.4)
	Non-anaemic	102 (35.4)	186 (64.6)	288 (64.6)
Education level	≤ Primary	44 (40.0)	66 (60.0)	110 (28.1)
	≥ Secondary	100 (35.4)	181 (64.6)	281 (71.9)
Employment status	Unemployed	96 (36.8)	165 (63.2)	261 (66.6)
	Employed	48 (36.6)	83 (63.4)	131 (33.4)
Marital status	Not living with a partner	34 (29.6)	81 (70.4)	115 (61.6)
	Living with a partner	75 (44.4)	94 (55.6)	169 (38.4)
Household contact with TB	Yes	14 (58.3)	10 (41.7)	24 (6.2)
	No	129 (35.3)	236 ((64.7)	365 (93.8)
Smoking	No	101 (35.0)	188 (65.0)	289 (73.7)
	Yes	43 (41.8)	60 (58.2)	103 (26.3)
Alcohol consumption	No	87 (35.2)	160 (64.8)	247 (62.7)
	Yes	57 (38.8)	90 (61.2)	147 (37.3)
Household ventilation	Always/Only when warm	13 (50.0)	13 (50.0)	26 (6.6)
(sleep with window open)	Never/No windows	131 (35.6)	237 (64.4)	368 (93.4)
Household SES	Low	43 (29.9)	101 (70.1)	144 (34.0)
	Medium	50 (33.8)	98 (66.2)	148 (35.0)
	High	51 (38.9)	80 (61.1)	131 (31.0)
Household exposure to	No	115 (36.4)	200 (63.6)	315 (79.9)
secondary smoking	Yes	29 (36.7)	50 (63.3)	79 (20.1)
Household number of	<3	102 (31.3)	224 (68.7)	326 (73.1)
rooms	≥3	51 (42.5)	69 (57.5)	120 (26.9)
Household density	Low (<300/km^2^)	53 (34.0)	103 (66.0)	156 (35.0)
	Medium (300–600/km^2^)	56 (38.9)	88 (61.1)	144 (32.3)
	High (>600/km^2^)	44 (30.1)	102 (69.9)	146 (32.7)

### Distribution of TST results, LTBI Prevalence and Annual Risk of infection

**Fig. 2:**
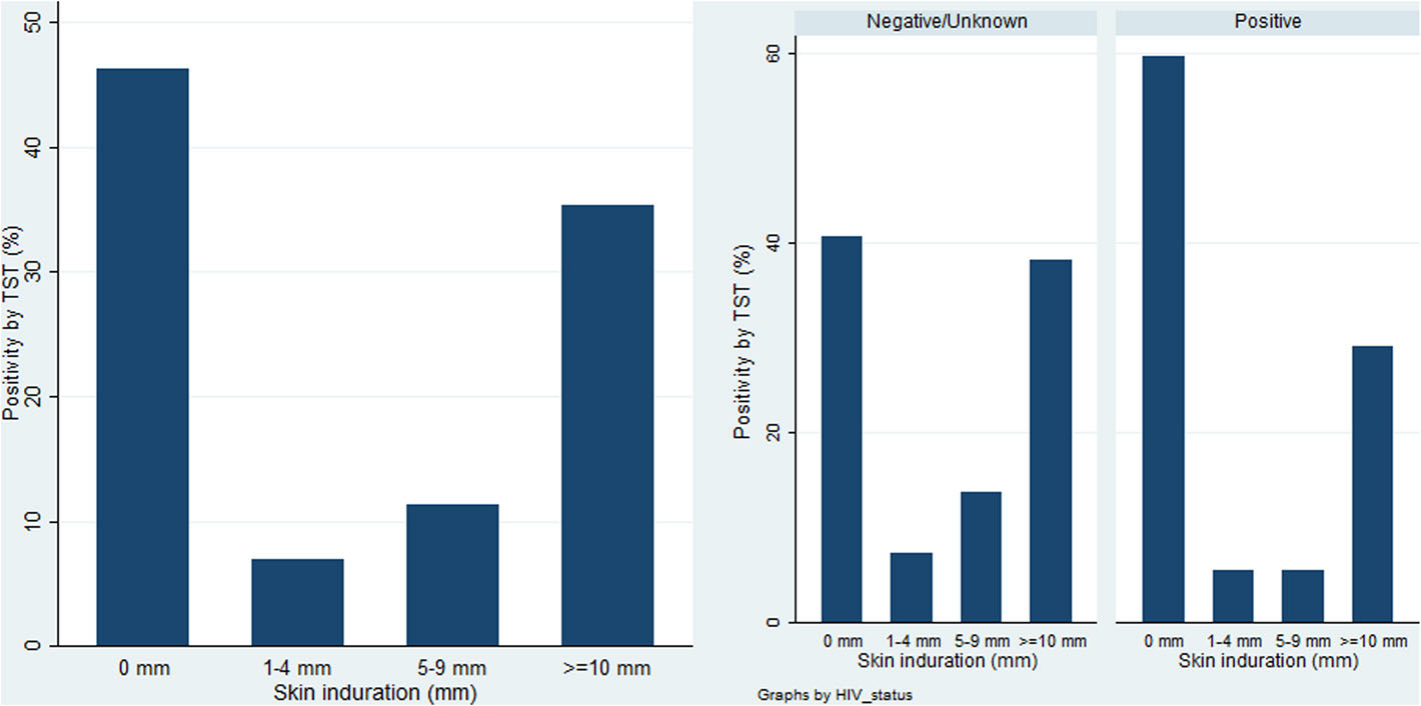
Distribution of TST induration diameter among the study participants and by HIV status

The frequency distributions of the indurations are shown in Fig. 2. Using HIV-specific definitions for LTBI, the overall prevalence of LTBI was 34 % [95 % CI, 30–39 %]. LTBI prevalence increased with age, from 19 % in the 0–14 age group to 45 % in the 45 and older age group (*p* = 0.002), was higher in women (37 %) than men (32 %) (*p* = 0.273), but similar in HIV positive (36 %) and HIV negative (32 %) participants (*p* = 0.553) (Table 2). Based on changes in TST prevalence with age among children age 0 to 15 years, the ARI was estimated at 3.1 % (95 % CI: 2.1–5.2).

**Table 2:** Estimated prevalence of infection by age, sex, and HIV status

Characteristics	Mean age Years	Prevalence, % % (95 % CI)	*p*-value
Overall	32.2	34.3 (30.0–38.8)	
Age group years			
0–14	6.2	18.8 (10.0–32.5)	0.002
15–24	20.7	26.3 (17.6–37.4)	
25–34	29.5	30.6 (23.7–38.8)	
35–44	39.1	45.1 (34.6–56.1)	
45+	53.4	45.2 (35.3–55.4)	
Sex			
Male	33.3	37.1 (30.3–44.8)	0.273
Female	31.8	32.1 (26.7–37.9)	
HIV status			
Positive	38.1	32.4 (22.5–44.2)	0.553
Negative	32.7	36.1 (31.0–41.5)	

### Factors associated with LTBI at individual and household level

In univariable logistic regression, age showed a strong association with LTBI with increasing odds of LTBI for every year increase in age (OR = 1.17, 95 % CI = 1.08–1.26) (Table 3). Other variables associated with LTBI were marital status, with individuals living with a partner being twice as likely to have LTBI compared with those living without a partner (OR = 2.00, 95 % CI: 1.06–3.80); history of household contact with TB, with those reporting such history being twice as likely to have LTBI compared with those not in household contact with a TB case (OR = 2.33, 95 % CI: 1.03–5.28); and number of room in the house, with people living in dwellings with 3 or more rooms being more likely to have LTBI compared to people living in dwellings with less than 3 rooms (OR = 1.62, 95 % CI: 1.05–2.50). People of the highest tertile of SES were 1.5 times more likely to have LTBI, but the 95 % CI crossed 1 (95 % CI 0.91–2.47). In multivariable logistic regression, age (OR = 1.03, 95 % CI = 1.01–1.05), gender (OR = 1.77, 95 % CI = 1.10–2.86), marital status (OR = 2.00, 95 % CI = 1.13–3.54) and living in a household that belong s to the highest tertile SES of the community (OR 2.11, 95 % CI 1.04–4.31) were independently associated with a diagnosis of LTBI.

**Table 3:** Logistic regression analysis of risk factors associated with LTBI

Characteristics		Unadjusted odds ratio (95 % CI)	Adjusted odds ratio (95 % CI)
Individual-level characteristics			
Age in years		1.17 (1.08–1.26)	1.03 (1.01–1.05)
Sex	Female	1.00	1.00
	Male	1.25 (0.84–1.86)	2.70 (1.55–4.70)
HIV status	Negative	1.00	
	Positive	0.85 (0.49–1.46)	
BMI categories	Normal/Underweight	1.00	
	Overweight	1.33 (0.80–2.19)	
	Obese	0.97 (0.60–1.59)	
Anaemia	Non-anaemic	1.00	
	Anaemic	0.87 (0.58–1.31)	
Education level	≤ Primary	1.00	
	≥ Secondary	0.83 (0.53–1.30)	
Employment status	Unemployed	1.00	
	Employed	0.99 (0.64–1.54)	
Marital status	Not living with a partner	1.00	1.00
	Living with a partner	1.90 (1.50–3.14)	2.00 (1.13–3.54)
Household contact with TB	No	1.00	1.00
	Yes	2.33 (1.03–5.28)	2.27 (0.76–6.82)
Smoking	No	1.00	
	Yes	1.33 (0.84–2.11)	
Alcohol consumption	No	1.00	
	Yes	1.16 (0.76–1.78)	
Household- and neighbourhood-level characteristics			
Household exposure to	No	1.00	
secondary smoking	Yes	1.01 (0.61–1.69)	
Household number of	<3	1.00	
rooms	≥3	1.62 (1.05–2.50)	
Household ventilation	Always/Only when warm	1.00	
(sleep with window open)	Never/No windows	1.37 (0.29–6.53)	
Household SES	Low	1.00	1.00
	Medium	1.20 (0.73–1.96)	1.73 (0.85–3.52)
	High	1.50 (0.91–2.47)	2.11 (1.04–4.31)
Household density	Low (<300/km^2^)	1.00	
	Medium (300–600/km^2^)	1.24 (0.77–1.98)	
	High (>600/km^2^)	0.84 (0.52–1.36)	

### Factors associated with LTBI at neighbourhood level

None of the neighbourhood level factors were associated with LTBI. The multilevel “null” model showed that ICC was 0.01032 (*p* = 0.4005), meaning that only 1 % of the variance in LTBI was explained by differences in neighbourhood factors.

## Discussion

The burden of LTBI in this urban informal settlement community of northern Johannesburg, South Africa, was high with an overall prevalence of 34.3 % and an annual risk of infection of 3.1 %. Risk factors independently associated with LTBI prevalence were older age, male gender, living with a partner, and high SES.

While the LTBI burden observed was high, the 34.3 % prevalence was lower than that the LTBI burden that has been observed in the few prior population-based studies previously performed in urban townships. In a Peruvian shantytown and a Ugandan urban population, the LTBI prevalence was higher, with half of all residents were living with LTBI (52 %; 95 % CI: 48–57 in Peru and 49 %; 95 % CI: 44–55 in Uganda) [31, 32]. A study of 8 South African urban communities however showed that LTBI prevalence among household contacts can be highly variable between communities in the same region, as they documented a range of LTBI prevalence from 24 to 77 % [33]. The ARI in our study fell within the range of ARI estimates from prior South African studies (2.8–5.8 %) [15, 34]. Taking together, these results suggest that the LTBI prevalence in urban settlements is high, but shows substantial variation.

Exposure to a household TB case is well established risk factor for LTBI [31, 35, 36], resulting in a large proportion of LTBI among children and young adults being due to household exposure to TB [37]. In our study, exposure to a household TB case was not significantly associated with LTBI. This may be due to relatively small sample size of children under 12 years of age in our study population. The increasing prevalence of LTBI with age reflects the cumulative exposure to TB through social interaction in high TB burden settings [38–42] and is consistent with findings of other LTBI studies in urban populations [15–17, 32, 33]. Data on the association between male gender and increased LTBI prevalence are conflicting. A higher LTBI prevalence among males was also observed in a rural area of Ethiopia [43] and a Peruvian peri-urban shantytown [31] but not in an urban population in Ugandan [32] nor in prior South Africa studies [16, 33, 44]. Being male was a strong predictor of LTBI on our study. Recent evidence suggests that social mixing and interaction vary significant by age and gender [45]. The higher rate of LTBI in urban males we observed may be due to the high risk of TB transmission in social gathering places, such as informal alcohol drinking establishments (shebeens) [39, 46], which are more frequented by men than women.

HIV infection was common (18 %) but not associated with LTBI prevalence in this population. Other LTBI prevalence studies in high HIV burden settings have reported similar observations [32, 47]. The lack of association between HIV and LTBI may be due poor sensitivity of TST in HIV-infected individuals [48], however we addressed this by decreasing the TST cut-off to 5 mm [49]. In addition, some other risk factors such as smoking and exposure to household secondary smoking [50, 51] were not associated with LTBI prevalence in our study. The smoking and exposure to secondary smoking have been found to be associated with increased odds of LTBI in low incidence settings [52, 53]. However, studies conducted in Africa have reported conflicting results [32, 33, 44–55]. Our findings are similar to results observed in studies conducted in Uganda, Zambia and South Africa [32, 33].

TB disease has clearly been established a as disease of poverty [56, 57]. It is therefore surprising that we observed a higher LTBI prevalence among people with higher household SES. An association of higher SES rather than lower SES associated with higher LTBI prevalence was also observed in a study in Zambia [47], and in a population-based multicentre study in China [58]. Another study in South Africa found employment not unemployment (which is one of the indicators for lower SES) was associated with higher LTBI prevalence [54]. Taken together, these findings suggest that SES may have a differential effect on the risk of LTBI acquisition and risk of progression from infection to active TB disease. Boccia et al suggested that “it is possible that, especially in urban settings, higher SEP is associated with housing characteristics that reduce ventilation and life-styles that increase social mixing and therefore the likelihood of contact between cases and susceptible people. We could not find an association between ventilation and LTBI, and higher SES was not associated with poorer ventilation in our sample. Given that we did not assess use of public transportation or social mixing [39, 59, 60] in our study we could not assess whether these factors can explain the observation of higher LTBI prevalence in people of higher SES within urban settlements. These hypotheses thus warrant further in-depth investigations.

Our study had many strengths, including the population-based design with geographically weighted random sampling of the general population, including people living with and without HIV and both adults and children, and a standardized approach to define SES tertiles. Our study does have some limitations. First, the cross-sectional nature of the study does not allow for establishment of temporality or causality between LTBI and associated factors. Second, small variability at the neighbourhood level may have been due to the sparsity of level 2 clusters with only 20 neighbourhoods (level 2 clusters), smaller than recommendation of 50 level 2 clusters [61]. Thus, fitting a multilevel logistic regression model was thus not indicated for the analysis of our data. Third, even though some of well-known risk factors such occupation, crowding, and ventilation were not measured, the proxy measures of these factors were not associated with LTBI. BCG vaccination status, which can reduce the specificity of TST, was also not documented [62, 63]. Fourth, the ethics committee restricted the placement of TST only to HIV positive individuals and children under 5 years old in October 2014. However, changes regarding with restriction of TST to HIV positive individuals is negligible since only 16 participants were included in the study after the restriction was introduced. Excluding these 16 participants does not change the results. We therefore feel confident that change (imposed by the ethics committee) did not affect the results. Furthermore, the sample size was relatively small, especially for children under 12 years of age since we did not made more attempts to find this group of participants if they were not at home during interview of the adult participants. Finally, as 19 % of the targeted household were not enrolled due to failure to find someone at home despite multiple attempts or refusal to participate, our aim to enrol a representative sample of the population may not have been fully achieved.

## Conclusion

The prevalence of LTBI and the annual risk of infection with *M. tuberculosis* is high in urban populations, especially in men, but independent of HIV infection status The unexpected association between higher LTBI and higher household SES suggest that the differential association between SES as risk factors for acquisition of TB infection and progression from LTBI to active disease is not yet fully understood. A better understanding of individual, household and community-level risk factors for LTBI will be important for the development of efficient, targeted LTBI interventions in high TB burden settings.

## Abbreviations

95 % CI: 95 % confidence interval
ARI: Annual risk of infection
BCG: Bacillus Calmette–Guérin
GPS: Geographic positioning system
HIV: Human immunodeficiency virus
ICC: Intraclass correlation coefficient
LTBI: Latent tuberculosis infection
OR: Odds ratio
PPD: Purified protein derivative
SES: Socioeconomic status
TB: Tuberculosis
TST: Tuberculin skin test
